# Draft Genome Sequence of Marine Sponge Symbiont *Pseudoalteromonas luteoviolacea* IPB1, Isolated from Hilo, Hawaii

**DOI:** 10.1128/genomeA.01002-16

**Published:** 2016-09-22

**Authors:** Francis E. Sakai-Kawada, Christopher J. Yakym, Martin Helmkampf, Kehau Hagiwara, Courtney G. Ip, Brandi J. Antonio, Ellie Armstrong, Wesley J. Ulloa, Jonathan D. Awaya

**Affiliations:** a Department of Molecular Biosciences and Bioengineering, University of Hawaii at Manoa, Honolulu, Hawaii, USA; b Tropical Conservation Biology and Environmental Science, University of Hawaii at Hilo, Hilo, Hawaii, USA; c Department of Pharmaceutical Sciences, Daniel K. Inouye College of Pharmacy, University of Hawaii at Hilo, Hilo, Hawaii, USA; d Biology Department, University of Hawaii at Hilo, Hilo, Hawaii, USA

## Abstract

We report here the 6.0-Mb draft genome assembly of *Pseudoalteromonas luteoviolacea* strain IPB1 that was isolated from the Hawaiian marine sponge *Iotrochota protea*. Genome mining complemented with bioassay studies will elucidate secondary metabolite biosynthetic pathways and will help explain the ecological interaction between host sponge and microorganism.

## GENOME ANNOUNCEMENT

Marine sponges are known to harbor diverse microbial communities. The species composition of these microbial communities is dependent on temporal and geographic factors (1–3). Many of the associated microorganisms are capable of producing bioactive secondary metabolites, which contribute to the ecological success of the host organism (4, 5). *Pseudoalteromonas luteoviolacea* strain IPB1 is a Gram-negative marine bacterium that was isolated from a Hawaiian marine sponge, *Iotrochota protea*, found in Puhi Bay (Hilo, HI), and identified from 16S rRNA sequencing and phylogenetic analysis. To our knowledge, this is the first published genome of *P. luteoviolacea* found within a marine sponge host (6, 7).

Genomic DNA of strain IPB1 was isolated using the UltraClean microbial DNA isolation kit (Mo Bio Laboratories, Inc.). A genome library was prepared using a Fast DNA fragmentation and library prep set for Ion Torrent (New England BioLabs), selected to a target length of 480 bp. An Ion Torrent library quantification kit (Kapa Biosystems) and a high-sensitivity DNA kit (Agilent) were used to determine the library dilution factor and assess the library size distribution, respectively. Emulsion PCR was performed using the Ion PGM Hi-Q OT2 kit-400 on an Ion OneTouch 2 system. The percent templated Ion Sphere particles for unenriched samples was measured with the Ion Sphere quality control kit on the Qubit fluorometer 3.0. Template enrichment was performed on Ion OneTouch enrichment system. The sample was loaded into an Ion 318 Chip version 2 and sequenced using the Ion PGM Hi-Q sequencing kit on the Ion PGM system.

The sequencing yielded 5,378,737 reads, with an average read length of 306 bp, totaling 1.65 Gb. Quality assessment of with the SPAdes software assembly resulted in a final genome assembly of 91 contigs, with a total size of 6,068,981 bp (265-fold coverage), an *N*_50_ contig length of 539,200 nucleotides, and a mean G+C content of 42.7%. The draft genome was annotated by the Rapid Annotations using Subsystems Technology (RAST) server and NCBI Prokaryotic Genome Annotation Pipeline (PGAP), which predicted 4,885 gene-coding sequences and 128 RNAs (73 tRNAs and 51 rRNAs) (8, 9). Secondary metabolite gene clusters were characterized using antiSMASH and PRISM (10–14). Further genome analysis and manipulation will be performed to determine the biosynthetic pathways involved in secondary metabolite production. By understanding the function of *P. luteoviolacea* IPB1, it will help toward elucidating its ecological role within the Hawaiian marine sponge host, *I. protea*.

### Accession number(s).

The annotated draft genome sequence was deposited in DDBJ/EMBL/GenBank under accession no. MAUJ00000000. The version described in this paper is version MAUJ00000000.1.
